# Sacrocolpopexy tends to be superior to transvaginal mesh surgery

**DOI:** 10.1111/1471-0528.16407

**Published:** 2020-08-03

**Authors:** Nikolaus Veit‐Rubin, Rufus Cartwright

**Affiliations:** ^1^ Department of Obstetrics and Gynaecology Medical University of Vienna Vienna Austria; ^2^ Oxford University Hospitals NHS Foundation Trust Oxford UK

The use of synthetic mesh prostheses to treat pelvic organ prolapse remains highly controversial. Despite complete restriction on vaginally placed mesh in Australia and New Zealand (2017), the UK (2018) and the USA (2019), transvaginal mesh (TVM) prolapse repair remains widely used in many countries, including China and most of East Asia. Sacrocolpopexy is widely considered as the reference standard for surgical correction of vaginal vault prolapse (Maher et al. *Cochrane Database Syst Rev* 2016;11:CD004014), whereas hysteropexy is an increasingly popular option for uterine prolapse (Meriwether et al. *Am J Obstet Gynecol* 2018;219:129–46.e2.). Both sacrocolpopexy and hysteropexy are considered safe and effective when performed laparoscopically. Although the comparison addressed in this systematic review (Zhang CY et al, *BJOG 2021*; 128:14–23) between sacrocolpopexy or hysteropexy and TVM for prolapse is not relevant in many settings, it remains of great importance for many urogynaecologists worldwide.

On the basis of four randomised controlled trials (RCTs), and consistent findings from six nonrandomised studies, the authors assert similar subjective success rates for both techniques. Abdominal mesh appears preferable to TVM surgery for mesh‐related complication rates, and for de novo dyspareunia. Although sexual function itself, as distinct from dyspareunia, could not be meta‐analysed as a separate outcome, it is noteworthy that the evidence favours abdominal mesh for postoperative total vaginal length, and the Pelvic Organ Prolapse Quantification System point C, which could be an indicator for improved sexual function. Overall, the evidence is of low or very low quality, with generally wide confidence intervals, statistical and clinical heterogeneity in the included RCTs and concerns about indirectness. The findings of the review, and the applicability of the evidence summaries for current practice, have to be understood in the context of an evolving regulatory environment with changing availability of different mesh products and limited evidence for newer lightweight meshes.

The authors conclude that additional high‐quality RCTs are needed to further evaluate the functional outcomes and complications of the two types of surgical procedure. There clearly will be little appetite for such trials in the UK, USA or Australasia. Most surgeons worldwide are likely to continue to rely on techniques with better risk profile than TVM, including vaginal native tissue repair and laparoscopic sacrocolpopexy and hysteropexy. There is a trend to offer these latter procedures to sexually active women with a long remaining life expectancy.

Surgeons who do continue to offer TVM need to submit data to high‐quality device registries about long‐term risk of mesh complications, which can occur many years into follow up. There has been significant improvement in the quality of TVM material, and TVM surgery may continue to develop within the framework of controlled clinical trials. In some jurisdictions, it may retain a role as a second‐line option for complex and recurrent cases. However, the exact indications for each pelvic organ prolapse repair technique remain uncertain, with progress likely to be hampered by a lack of standardisation in how they are performed. Decisions in each case need to take account of a woman’s individual goals and the specific risks of surgical complications, as well as the surgeon’s proficiency for each technique.

## Disclosure of interests

No conflict of interests to declare. Completed disclosure of interests forms are available to view online as supporting information.

## Supporting information

Supplementary Material

Supplementary Material

